# The Antiviral Effect of Panax Notoginseng Polysaccharides by Inhibiting PRV Adsorption and Replication In Vitro

**DOI:** 10.3390/molecules27041254

**Published:** 2022-02-13

**Authors:** Changchao Huan, Ziyan Zhou, Jingting Yao, Bo Ni, Song Gao

**Affiliations:** 1 Institutes of Agricultural Science and Technology Development, College of Veterinary Medicine, Yangzhou University, Yangzhou 225009, China; changchaohuan@yzu.edu.cn (C.H.); yzdxzzy@163.com (Z.Z.); yaojingting201609@163.com (J.Y.); 2 Jiangsu Co-innovation Center for Prevention and Control of Important Animal Infectious Diseases and Zoonoses, Yangzhou 225009, China; 3 Key Laboratory of Avian Bioproduct Development, Ministry of Agriculture and Rural Affairs, Yangzhou 225009, China; 4 China Animal Health and Epidemiology Center, Qingdao 266000, China; nibo@cahec.cn; 5 Institutes of Agricultural Science and Technology Development, Yangzhou University, Yangzhou 225009, China

**Keywords:** Panax notoginseng polysaccharides, pseudorabies virus (PRV), antiviral, infection, adsorption, replication

## Abstract

Porcine pseudorabies (PR) is an important infectious disease caused by pseudorabies virus (PRV), which poses a major threat to food safety and security. Vaccine immunization has become the main means to prevent and control the disease. However, since 2011, a new PRV variant has caused huge economic losses to the Chinese pig industry. Panax notoginseng polysaccharides have immunomodulatory activity and other functions, but the antiviral effect has not been reported. We studied the anti-PRV activity of Panax notoginseng polysaccharides in vitro. A less cytopathic effect was observed by increasing the concentration of Panax notoginseng polysaccharides. Western blot, TCID_50_, plaque assay, and IFA revealed that Panax notoginseng polysaccharides could significantly inhibit the infectivity of PRV XJ5 on PK15 cells. In addition, we also found that Panax notoginseng polysaccharides blocked the adsorption and replication of PRV to PK15 cells in a dose-dependent manner. These results show that Panax notoginseng polysaccharides play an antiviral effect mainly by inhibiting virus adsorption and replication in vitro. Therefore, Panax notoginseng polysaccharides may be a potential anti-PRV agent.

## 1. Introduction

Pseudorabies (PR) is a disease, caused by pseudorabies virus (PRV), which is highly infectious and has a high mortality rate in the swine industry, and transmission mainly occurs through oral and nasal secretions, but it also can be transmitted through the placenta and aerosols, and via sexual intercourse [1]. Piglets and pregnant sows are the major victims: Younger, suckling swine’s clinical symptoms mainly include neurological signs and a high risk of death; as for pregnant swine, PRV can result in abortion or delivery of stillborn or weakened piglets that die shortly after birth [2]. It can cause the death of piglets, as well as miscarriage of pregnant sows and stillbirth, thus bringing unpredictable economic losses to the pig industry [3]. With the wide application of PRV vaccine, the epidemic situation has been effectively controlled. However, since 2011, serious PR has broken out in many places in China, and the positive rate is increasing year by year [4]. Because the newly isolated strains have varying degrees of variation and virulence, which can cause in infected pigs highly pathogenic and differing degrees of clinical symptoms [5,6]. In addition, PRV could infect humans, and it was isolated from an acute human encephalitis case [7,8,9]. Therefore, it is particularly urgent and important to prevent and control PRV infection.

As a traditional medicine, ginseng has been used for thousands of years in Eastern countries. Although there are many known ginseng species that exist, Panax notoginseng is one particular species that has a wide function in medicinal use. It has been reported to have a range of pharmacological effects, such as central nervous and immune system modulation, protection of the cardiovascular system, and antioxidant, antidiabetic, antitumor, and antiaging activities [10,11]. It also has an important role in promoting blood circulation and removing blood stasis; reducing swelling; and relieving pain; it also has anti-inflammation and anti-tumor properties [12,13]; improves immunity; and so on [14,15,16,17]. Panax notoginseng polysaccharides are a main, active component of Panax notoginseng, which have obvious inhibitory and analgesic effects on the central nervous system [18]. They can promote and strengthen the function of the reticuloendothelial system, delay aging, resist microwave irradiation, and improve immunity, and they can repair bone defects and promote recovery from trauma [19]. Studies have shown that Panax notoginseng has antiviral activity against influenza A virus and human immunodeficiency virus-1 [20,21]. However, the effect of Panax notoginseng polysaccharides on PRV has not been reported. Therefore, we studied the antiviral effect of Panax notoginseng polysaccharides on PRV infection in vitro.

## 2. Results

### 2.1. Panax Notoginseng Polysaccharides Have No Cytotoxic Effect on PK15 Cells

**Figure 1:**
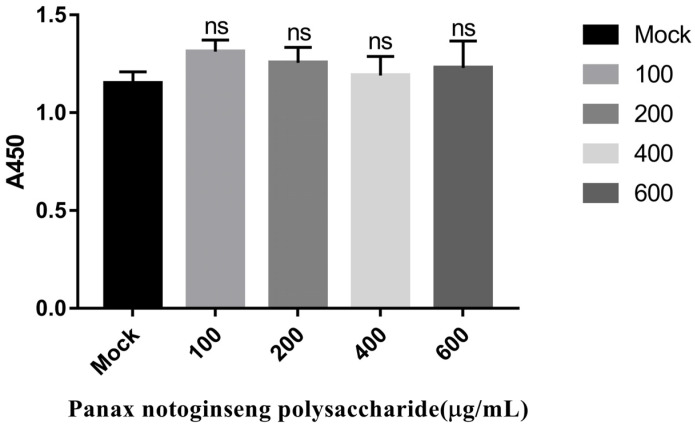
Panax notoginseng polysaccharides have no cytotoxic effect on PK15 cells. PK15 cells were pretreated with different concentrations of Panax notoginseng polysaccharides for 24 h at 37 °C, and then 10 μL Enhanced Cell Counting Kit-8 was added to each hole for 1 h at 37 °C. We measured absorbance at 450 nm. All data are presented as means ± SD and were analyzed by GraphPad Prism software (GraphPad Software, San Diego, CA, USA) using one-way ANOVA (ns = not significant).

In order to explore the effect of Panax notoginseng polysaccharides on PK15 cells, we checked the cell growth by Enhanced Cell Counting Kit-8. Figure 1 reveals that Panax notoginseng polysaccharides have no cytotoxic effect on PK15 cells. 

### 2.2. Panax Notoginseng Polysaccharides Can Inhibit PRV Infection in PK15 Cells 

**Figure 2:**
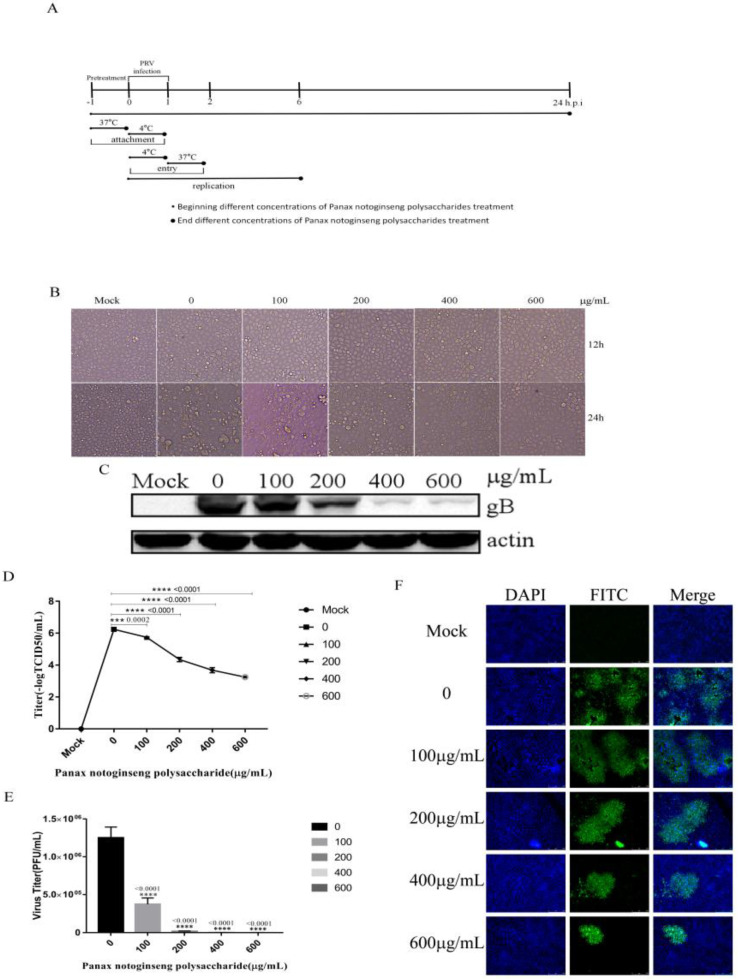
Panax notoginseng polysaccharides inhibited PRV infection in PK15 cells. PK15 cells were pretreated with 100, 200, 400, and 600 μg/mL Panax notoginseng polysaccharides for 1 h before being infected with PRV XJ5 (MOI = 0.1). Then, infected cells were cultured with 100, 200, 400, and 600 μg/mL Panax notoginseng polysaccharides. (**A**) The procedure for antiviral activity assay, adsorption assay, entry assay, and replication assay; (**B**) Infected cells were observed at 12 and 24 hpi; (**C**) At 24 hpi, gB protein was detected by Western blot; (**D**,**E**) Virus titer was evaluated by 50% tissue culture infective dose TCID_50_ and plaque assay. All data are presented as means ± SD and were analyzed by GraphPad Prism software (GraphPad Software, San Diego, CA, USA) using one-way ANOVA (*** *p* = 0.002, **** *p* < 0.0001); (**F**) IFA for internalized virus was performed.

To explore the effect of Panax notoginseng polysaccharides on PRV infection, we outline the procedure for antiviral activity assay, adsorption assay, entry assay, and replication assay (Figure 2A). PK15 cells were pretreated with different concentrations of Panax notoginseng polysaccharides (100 μg/mL, 200 μg/mL, 400 μg/mL, and 600 μg/mL) for 1 h, and were infected with PRV XJ5 (MOI = 0.1) with different concentrations of Panax notoginseng polysaccharides for 12 and 24 h. Figure 2B shows that the obvious cytopathic effect induced by PRV XJ5 (MOI = 0.1) can be inhibited by Panax notoginseng polysaccharides. We used Western blot analysis to analyze the expression of PRV gB protein in cells. The results showed that the expression of PRV gB protein decreased significantly with the increase in the concentration of Panax notoginseng polysaccharides with an inhibition rate of 98.7% at 600 μg/mL Panax notoginseng polysaccharides (Figure 2C). The culture supernatant of PK15 cells treated with Panax notoginseng polysaccharides was collected, and the virus titer was determined by TCID50 and plaque assay. The results showed that Panax notoginseng polysaccharides reduced the production of virions in a dose-dependent manner (Figure 2D,E). In addition, IFA confirmed that Panax notoginseng polysaccharides significantly inhibited PRV infection with an inhibition rate of 93.6% at 600 μg/mL Panax notoginseng polysaccharides (Figure 2F). These results showed that Panax notoginseng polysaccharides could inhibit PRV infection.

### 2.3. Panax Notoginseng Polysaccharides Inhibit the Adsorption and Entry of PRV to PK15 Cells

**Figure 3:**
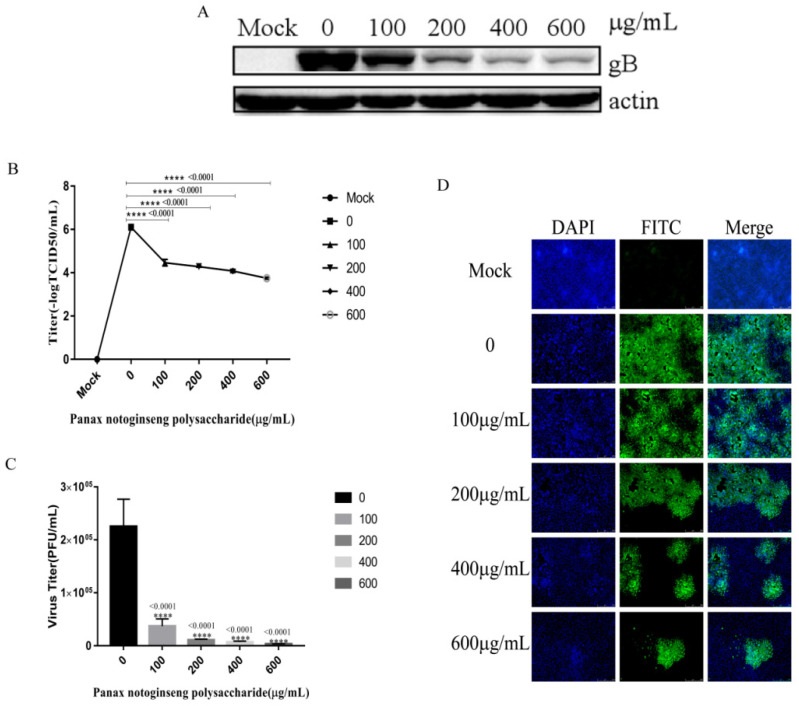
Panax notoginseng polysaccharides prevented pseudorabies virus (PRV) adsorption and entry to PK15 cells. PK15 cells were pretreated with 100, 200, 400, and 600 μg/mL Panax notoginseng polysaccharides for 1 h before cells were infected with PRV XJ5 (MOI = 0.1) at 4 °C for 1 h. Cells were incubated with 100, 200, 400, and 600 μg/mL Panax notoginseng polysaccharides at 37 °C for 1 h. (**A**) At 24 hpi, Western blot detected PRV gB protein expression; (**B**,**C**) viral titers were detected by TCID_50_ and plaque assay_._ All data are presented as means ± SD and were analyzed by GraphPad Prism software (GraphPad Software, San Diego, CA, USA) using one-way ANOVA (**** *p* < 0.0001); (**D**) IFA for internalized virus was performed.

We performed the experiment according to Section 4.7. The results show that the expression of PRV gB protein decreased gradually with the increase of the concentration of Panax notoginseng polysaccharides (Figure 3A). At the same time, the supernatant of the cells infected for 24 h was collected, and the virus titer was determined. The results showed that the titer of PRV virus decreased significantly (Figure 3B,C). In addition, IFA analysis further proved that Panax notoginseng polysaccharides could decrease the number of cells infected with PRV XJ5 by 96.4% at 600 μg/mL Panax notoginseng polysaccharides (Figure 3D). These results showed that Panax notoginseng polysaccharides can inhibit the adsorption and entry of pseudorabies virus.

### 2.4. Panax Notoginseng Polysaccharides Inhibit the Adsorption of PRV to PK15 cells

**Figure 4:**
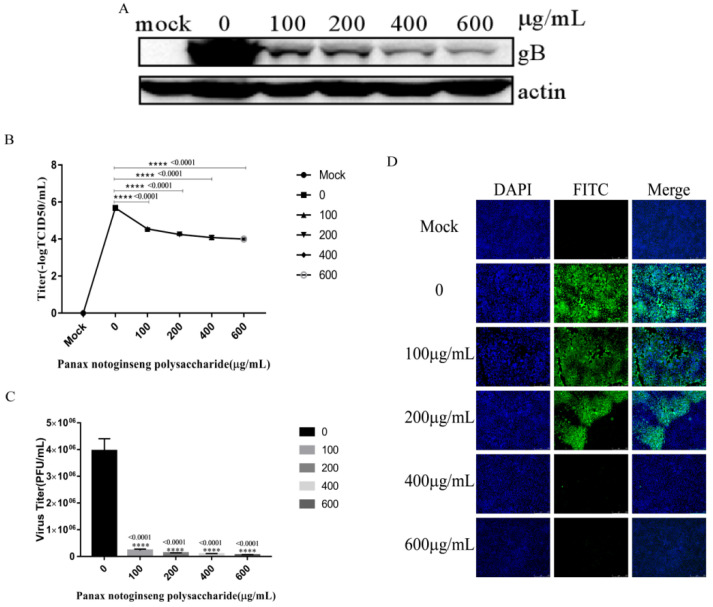
Panax notoginseng polysaccharides prevent pseudorabies virus (PRV) adsorption in PK15 cells. PK15 cells were pretreated with 100, 200, 400, and 600 μg/mL Panax notoginseng polysaccharides for 1 h, then infected with PRV XJ5 (MOI = 0.1) at 4 °C for 1 h in the presence of 100, 200, 400, and 600 μg/mL Panax notoginseng polysaccharides before the removal of the Panax notoginseng polysaccharides medium. Cells were cultured in Panax notoginseng polysaccharide-free 2% DMEM. (**A**) At 24 hpi, Western blot analysis detected PRV gB protein expression; (**B**,**C**) Viral titers were detected by TCID_50_ and plaque assay_._ All data are presented as means ± SD and were analyzed by GraphPad Prism software (GraphPad Software, San Diego, CA, USA) using one-way ANOVA (**** *p* < 0.0001); (**D**) IFA for internalized virus was performed.

We performed the experiment according to Section 4.8. The results of Western blot analysis showed that the expression of PRV gB protein decreased with the increase in the concentration of Panax notoginseng polysaccharides (Figure 4A). The supernatant of cells infected for 24 h was collected for virus titer determination, and the virus titer decreased gradually with the increase of the concentration of Panax notoginseng polysaccharides (Figure 4B,C). In addition, IFA revealed that Panax notoginseng polysaccharides could decrease the number of cells infected with PRV XJ5 by about 99.8% at 600 μg/mL Panax notoginseng polysaccharides (Figure 4D). These results confirmed that Panax notoginseng polysaccharides significantly decreased PRV attachment.

### 2.5. Panax Notoginseng Polysaccharide Can’t Inhibit the Entry of PRV to PK15 Cells

**Figure 5:**
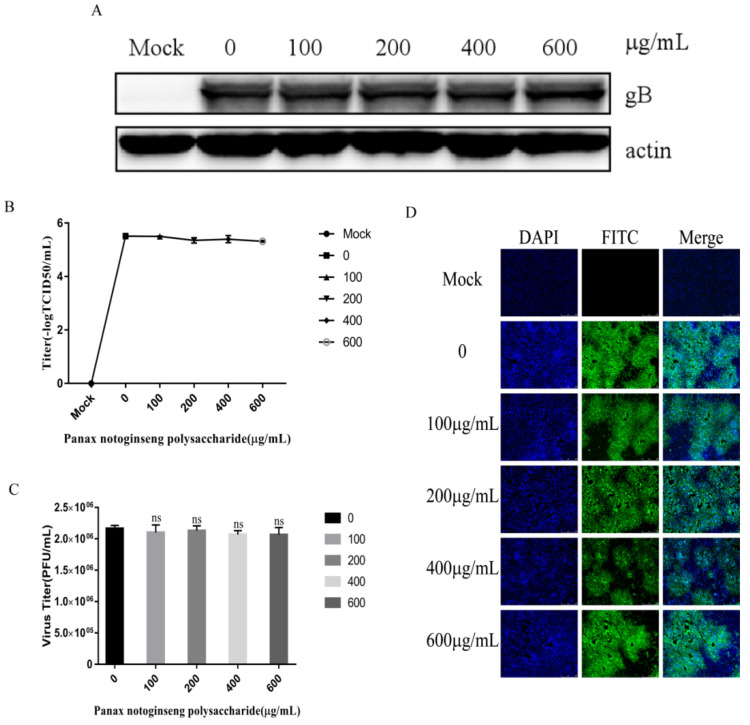
Panax notoginseng polysaccharides did not prevent pseudorabies virus (PRV) entry to PK15 cells. PK15 cells were infected with PRV XJ5 (MOI = 0.1) at 4 °C for 1 h, and then infected cells were treated with 100, 200, 400, and 600 μg/mL Panax notoginseng polysaccharides for 1 h at 37 °C, before removal of the Panax notoginseng polysaccharides medium. Cells were cultured in Panax notoginseng polysaccharide-free 2% DMEM. (**A**) At 24 hpi, Western blot analysis detected PRV gB protein expression; (**B**,**C**) Viral titers were detected by TCID_50_ and plaque assay_._ All data are presented as means ± SD and were analyzed by GraphPad Prism software (GraphPad Software, San Diego, CA, USA) using one-way ANOVA (ns = not significant); (**D**) IFA for internalized virus was performed.

We performed the experiment according to Section 4.9. The results of Western blot analysis, TCID_50_, plaque assay, and IFA showed that Panax notoginseng polysaccharides could not inhibit the entry of pseudorabies virus into PK15 cells (Figure 5A–D). Based on the above results, Panax notoginseng polysaccharides mainly inhibit the adsorption of PRV to PK15 cells.

### 2.6. Panax Notoginseng Polysaccharides Reduce the Replication of PRV in PK15 Cells

**Figure 6:**
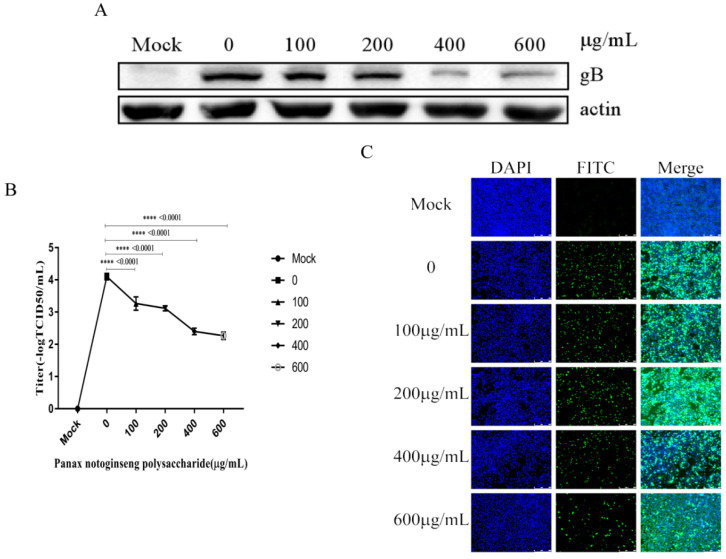
Panax notoginseng polysaccharides inhibited pseudorabies virus (PRV) replication in PK15 cells. PK15 cells were infected with PRV XJ5 (MOI = 0.1) at 37 °C for 1 h, and then infected cells were incubated with 100, 200, 400, and 600 μg/mL Panax notoginseng polysaccharides until harvest. (**A**) At 6 hpi, cells were collected to detect the expression of PRV gB protein by Western blot analysis; (**B**) Viral titers were detected by TCID_50_. All data are presented as means ± SD and were analyzed by GraphPad Prism software (GraphPad Software, San Diego, CA, USA) using one-way ANOVA (**** *p* < 0.0001); (**C**) IFA for internalized virus was performed.

We performed experiment according to Section 4.10. Western blot analysis was used to analyze the expression of viral protein gB at 6 hpi (Figure 6A). The supernatant of cells infected for 6 h was collected for virus titer determination, and the virus titer decreased gradually with the increase of the concentration of Panax notoginseng polysaccharides (Figure 6B). In addition, IFA revealed that Panax notoginseng polysaccharides could reduce the numbers of cells infected with PRV XJ5 (Figure 6C). These results indicated that Panax notoginseng polysaccharides could inhibit virus replication.

## 3. Discussion

Panax notoginseng is a perennial herb of Acanthopanax senticosus, which is a valuable traditional Chinese medicine with the functions of hemostasis, tonifying blood, promoting blood circulation, reducing swelling, and relieving pain. The polysaccharides in Panax notoginseng are extracted by researchers through a variety of methods [22,23]. Polysaccharides are an important active component of Panax notoginseng and have significant activity in immune regulation. Some studies have shown that a variety of polysaccharides can be isolated from Panax notoginseng roots, among which PNPSII can restore the proliferation of lymphocytes in immunosuppressive mice induced by cyclophosphamide in vivo [24].

At present, there are no related studies that report the inhibitory effect of Panax notoginseng polysaccharides on PRV. Our study revealed that Panax notoginseng polysaccharides inhibit PRV infection. We confirmed that Panax notoginseng polysaccharides could significantly inhibit PRV adsorption of PRV, but Panax notoginseng polysaccharides had no effect on the entry of PRV to PK15 cells. In addition, Panax notoginseng polysaccharides decreased the replication of PRV. Therefore, we confirmed that Panax notoginseng polysaccharides could significantly inhibit the infection of PRV XJ5 in vitro, mainly by inhibiting the adsorption and replication of PRV. However, their mechanism needs to be further studied. Panax notoginseng polysaccharides can not only protect against antioxidation and cerebral ischemic injury, but also inhibit tumor cells [25,26]. PBGA12, which is a Panax notoginseng polysaccharide, enhanced IFN-γ and TNF-α to stimulate the complement system [23]. Another polysaccharide fraction from notoginseng, Fr1MKOH, exhibited complement-fixing activity and a mitogenic effect on human polymorphonuclear neutrophils [27]. A polysaccharide from notoginseng decreased the amount of MDA and increased the activities of antioxidant enzymes, such as catalase (CAT), glutathione peroxidase (GSH-Px), and superoxide dismutase (SOD), by activating the transforming growth factor-β signaling pathway into H_2_O_2_-induced human dermal fibroblast cells [28]. The notoginseng polysaccharides inhibited a caspase-3 cascade, and regulated the alcohol dehydrogenase pathway, to protect from cerebral ischemia/reperfusion injury or alcoholic liver damage, respectively [29,30]. These functions may be the mechanism of Panax notoginseng polysaccharide against PRV infection.

We revealed that Panax notoginseng polysaccharides inhibited PRV infection by interfering with PRV adsorption and replication in vitro. But the mechanism of Panax notoginseng polysaccharide against PRV infection will need further research. In addition, the antiviral effect of Panax notoginseng polysaccharides in pigs will be further explored in order to reveal whether Panax notoginseng polysaccharides will be applied to pigs. 

In summary, we confirmed that Panax notoginseng polysaccharides inhibited PRV infection by interfering with PRV adsorption and replication in vitro, especially in the adsorption stage.

## 4. Materials and Methods

### 4.1. Cells

PK15 cells were separated from the porcine kidney and were cultured in Dulbecco’s Modified Eagle’s Medium (DMEM), supplemented with penicillin and streptomycin, fungizone, and 5% fetal bovine serum (Lonsa S711-001). Vero cells were cultured in DMEM supplemented with penicillin, streptomycin, fungizone, and 6% fetal bovine serum. All cells were cultured at 37 °C with 5% CO_2_.

### 4.2. Virus

PRV XJ5 was isolated by the Yangzhou University Infectious Diseases Laboratory and used in experiments. Virus stocks were stored at −70 °C. We determined the concentration of PRV virus by TCID_50_. The TCID_50_ value was calculated by the Reed-Muench method [31].

### 4.3. Reagents and Antibodies

Panax notoginseng polysaccharides [≥98% (uv)] obtained from Yangling Ciyuan Biotechnology Co., Ltd., China were diluted to stock solutions of 50 mg/mL with PBS and stored at −20 °C for all subsequent experiments. gB and PRV-positive sera were generated in our laboratory. β-actin antibody was obtained from TransGenBiotech (Beijing, China). FITC-conjugated goat anti-pig IgG antibody was purchased from Sigma-Aldrich (St. Louis, MO, USA). 4′,6-diamidino-2-phenylindole (DAPI) was purchased from Beyotime Biotechnology (Shanghai, China).

### 4.4. Cytotoxic effect of Panax Notoginseng Polysaccharide on PK15 Cells

PK15 cells were diluted with 5% DMEM and counted. The cells with a concentration of 5000 cells per well were placed in a 96-well plate and cultured at 37 °C with 5% CO_2_. When the cells grew to 70–80% density, the cells were used to perform experiments. PK15 cells were pretreated with different concentrations of Panax Notoginseng Polysaccharides (100 μg/mL, 200 μg/mL, 400 μg/mL, and 600 μg/mL) for 24 h at 37 °C, and then 10 μL Enhanced Cell Counting Kit-8 was added to each hole for 1 h at 37 °C. We measured absorbance at 450 nm.

### 4.5. Culture of Cells

The PK15 cells were diluted with 5% DMEM and counted. The cells with a concentration of 5 × 10^5^ cells per well were placed in a six-well plate and cultured at 37 °C with 5% CO_2_. When the cells grew to 70–80% density, the cells were used to perform experiments.

### 4.6. Effect of Panax Notoginseng Polysaccharide on Inhibition of PRV Infection in PK15 Cells

We pretreated the cells with different concentrations of Panax notoginseng polysaccharides (100 μg/mL, 200 μg/mL, 400 μg/mL, and 600 μg/mL) for 1 h. Then, the cells were infected with PRV XJ5 (0.1 multiplicity of infection (MOI)) for 1h with different concentrations of Panax notoginseng polysaccharides, and the cells were cultured in 2% DMEM-containing solution with a corresponding concentration of Panax notoginseng polysaccharides. The morphological changes and pathological changes of the cells were observed under microscope at 12 and 24 hpi. At 24 hpi, intracellular viral proteins were detected by Western blot analysis. Cell supernatants were used to measure virus titer by TCID_50_ and plaque assay. Detection of intracellular virus was performed via indirect immunofluorescent assay (IFA).

### 4.7. Effect of Panax Notoginseng Polysaccharide on Adsorption and Entry of Porcine Pseudorabies Virus

The cells were cultured with 1 mL serum-free DMEM, including the corresponding concentration of Panax notoginseng polysaccharides (100 μg/mL, 200 μg/mL, 400 μg/mL, and 600 μg/mL) at 37 °C for 1 h. Cells were infected with PRV XJ5 (0.1MOI) at cold serum-free DMEM with corresponding concentrations of Panax notoginseng polysaccharides (100 μg/mL, 200 μg/mL, 400 μg/mL, and 600 μg/mL) at 4 °C for 1 h. Cells were washed with cold PBS three times, and we added 1 mL 2% DMEM with corresponding concentrations of Panax notoginseng polysaccharides (100 μg/mL, 200 μg/mL, 400 μg/mL, and 600 μg/mL) at 37 °C for 1 h. Cells were washed with citric acid three times, and then washed with PBS three times. Cells were cultured with 2 mL 2% DMEM at 37 °C in a 5% CO_2_ incubator for 24 h. At 24 hpi, intracellular viral proteins were detected by Western blot analysis. Cell supernatants were used to measure virus titer by TCID_50_ and plaque assay. Detection of intracellular virus was performed via indirect immunofluorescent assay.

### 4.8. Effect of Panax Notoginseng Polysaccharide on Adsorption of PRV

The cells were cultured with 1 mL serum-free DMEM including the corresponding concentration of Panax notoginseng polysaccharides (100 μg/mL, 200 μg/mL, 400 μg/mL, and 600 μg/mL) at 37 °C for 1 h. Cells were infected with PRV XJ5 (0.1MOI) at cold serum-free DMEM with corresponding concentrations of Panax notoginseng polysaccharides (100 μg/mL, 200 μg/mL, 400 μg/mL, and 600 μg/mL) at 4 °C for 1 h. Cells were washed with cold PBS three times, and we added 2 mL 2% DMEM at 37 °C in a 5% CO_2_ incubator. At 24 hpi, intracellular viral proteins were detected by Western blot analysis. Cell supernatants were used to measure virus titer by TCID_50_ and plaque assay. Detection of intracellular virus was performed via indirect immunofluorescent assay.

### 4.9. Effect of Panax Notoginseng Polysaccharide on PRV Entry

The PK15 cells were diluted with 5% DMEM and counted. The cells with a concentration of 5 × 10^5^ cells per well were placed in a six-well plate and cultured at 37 °C with 5% CO_2_. After the cells grew to 70–80% density, the cells were washed three times with cold PBS, and cells were infected with PRV XJ5 (0.1MOI) at cold serum-free DMEM at 4 °C for 1 h. Cells were washed with cold PBS three times, and we added 1 mL 2% DMEM with corresponding concentrations of Panax notoginseng polysaccharides (100 μg/mL, 200 μg/mL, 400 μg/mL, and 600 μg/mL) at 37 °C for 1 h. Cells were washed with citric acid three times, and then washed with PBS three times. Cells were cultured with 2 mL 2% DMEM at 37 °C in 5% CO_2_ incubator for 24 h. At 24 hpi, intracellular viral proteins were detected by Western blot analysis. Cell supernatants were used to measure virus titer by TCID_50_ and plaque assay. Detection of intracellular virus was performed via indirect immunofluorescent assay.

### 4.10. Effect of Panax Notoginseng Polysaccharide on Replication of Porcine Pseudorabies Virus

PK15 cells were incubated with PRV XJ5 at 37 °C for 1 h. The cells were then washed three times with PBS and incubated with 2% DMEM containing 100 μg/mL, 200 μg/mL, 400 μg/mL, and 600 μg/mL Panax notoginseng polysaccharides. At 6 hpi, intracellular viral proteins were detected by Western blot analysis. Cell supernatants were used to measure virus titer by TCID_50_ assay. Detection of intracellular virus was performed via indirect immunofluorescent assay.

### 4.11. Western Blotting

The western blotting followed previous descriptions [32,33]. PRV gB and actin were checked via the above-mentioned primary antibody and a secondary antibody.

### 4.12. Virus Titer Assays

We used the 50% tissue culture infective dose (TCID_50_) assay to evaluate virus titers. Vero cells were seeded in 96-well plates with 6% FBS in DMEM. Virus samples were serially diluted from different dilution multiples in DMEM and used to inoculate cells at 37 °C for 1.5 h, and then removed from the virus–DMEM mixture. The infected cells were cultured in 2% DMEM at 37 °C with 5% CO_2_ for 72 h. Cytopathic effect (CPE) on Vero cells was counted to calculate the TCID_50_ by the Reed–Muench method.

### 4.13. Plaque Assays

Viral culture supernatants were diluted from 10^−1^ to 10^−6^ in DMEM, and then used to infect PK15 cells seeded in 6-well plates at 37 °C for 1–2 h before an overlay medium (2.5% low-melting-point agarose in DMEM medium containing 4% fetal bovine serum) was added to each well. PK15 cells were cultured at 37 °C with 5% CO_2_ for 3 days, after which they were stained with 0.5% crystal violet.

### 4.14. Indirect Immunofluorescent Assay (IFA)

PK15 cells were fixed with 4% paraformaldehyde for 15 min before incubation with 0.1% Triton X-100 for 10 min, and then blocked with 5% BSA at 37 °C for 30 min. After blocking, the cells were incubated with PRV-positive pig serum at 37 °C for 1 h, and cells incubated with FITC-conjugated goat anti-pig IgG antibody at 37 °C for 30 min. Then, cells were incubated with DAPI at 37 °C for 7 min before being observed under fluorescence microscopy. All images were taken at 100× magnification.

### 4.15. Statistical Analysis

All experiments were independently repeated at least three times, and all data are presented as means ± SD. The data were analyzed by GraphPad Prism software (GraphPad Software, San Diego, CA, USA) using one-way ANOVA. When * *p* < 0.05, ** *p* < 0.01, the differences were statistically significant.

## 5. Conclusions

The results showed that Panax notoginseng polysaccharides had an obvious inhibitory effect on PRV infection, and the inhibitory effect of Panax notoginseng polysaccharides on PRV was more obvious with increases in the concentration of Panax notoginseng polysaccharides. In addition, Panax notoginseng polysaccharides can inhibit the adsorption and replication of PRV in PK15. These results show that Panax notoginseng polysaccharides can be further developed into an antiviral agent against PRV infection.
